# Plasmablastic myeloma progressed to plasma cell leukaemia

**DOI:** 10.1002/jha2.331

**Published:** 2021-10-30

**Authors:** Ke Xu, Anna Childerhouse, Ahmed Al‐Hassani, Charalampia Kyriakou

**Affiliations:** ^1^ Department of Haematology University College London Hospitals NHS Foundation Trust, University College London London UK; ^2^ Specialist Integrated Haematology Malignancy Diagnostic Service Health Services Laboratories University College London Hospitals NHS Foundation Trust University College London London UK; ^3^ Department of Histopathology University College London Hospitals NHS Foundation Trust University College London London UK

**Keywords:** myeloma, plasmablastic myeloma, plasma cell leukaemia

A 37‐year‐old male presented with anaemia, acute kidney injury, hypercalcaemia. A full blood count showed haemoglobin 74 g/L, white blood cells 7 × 10^9^/L, neutrophils 5 × 10^9^/L, lymphocytes 2 × 10^9^/L, and platelets 200 × 10^9^/L. Kappa light chain was 1210 mg/L. Kappa/lambda ratio was 432. No paraprotein was detected. The computed tomography scan showed multiple lytic lesions in the axial and appendicular skeleton. The bone marrow smear was packed with medium to large‐sized blast‐like cells (Figure 1 top left image, May‐Grünwald‐Giemsa stain 100x objective). Trephine biopsy specimen was hypercellular, with an extensive diffuse interstitial infiltrate of mostly medium‐sized cells with high nuclear‐cytoplasmic ratio, coarse chromatin with sometimes small nucleoli (Figure 1 bottom left image, haematoxylin and eosin stain 40x objective). They were positive for CD138 (Figure 1 bottom right image, 40x objective), cyclin D1 and CD56 and they showed kappa light chain restriction. They were negative for CD79a, CD20, CD117, CD34, myeloperoxidase, CD123, CD71, Epstein‐Barr virus‐encoded RNA, CD30, human herpesvirus 8 and anaplastic lymphoma kinase. Targeted fluorescence in situ hybridization performed on CD138 enriched cells detected loss of 17p (*TP53*) and t(11;14) (*IGH‐MYEOV*) in 92% and 99% of CD138+ cells assessed. He was diagnosed with plasmablastic myeloma. He initially achieved a very good partial response following two cycles with a bortezomib (Velcade) thalidomide dexamethasone regimen. Unfortunately, with non‐compliance to chemotherapy, the disease progressed 4 months after diagnosis. Full blood count showed haemoglobin 97 g/L, white blood cells 29 × 10^9^/L, neutrophils 3.5 × 10^9^/L, lymphocytes 24.5 × 10^9^/L, and platelets 12 × 10^9^/L. Kappa light chain was 1081 mg/L. Kappa/lambda ratio was 284. The blood film showed pleomorphic atypical plasma cells (Figure 1 top right image, May‐Grünwald‐Giemsa stain 100x objective). Flow cytometry showed that 40% of total nucleated cells were cytoplasmic kappa‐restricted plasma cells. They were positive for CD138, CD38, CD56, and negative for CD19 and CD117. Features were indicative of plasma cell leukaemia. The disease was refractory to treatment and the patient died 1 week after diagnosis of plasma cell leukaemia.

**FIGURE 1:**
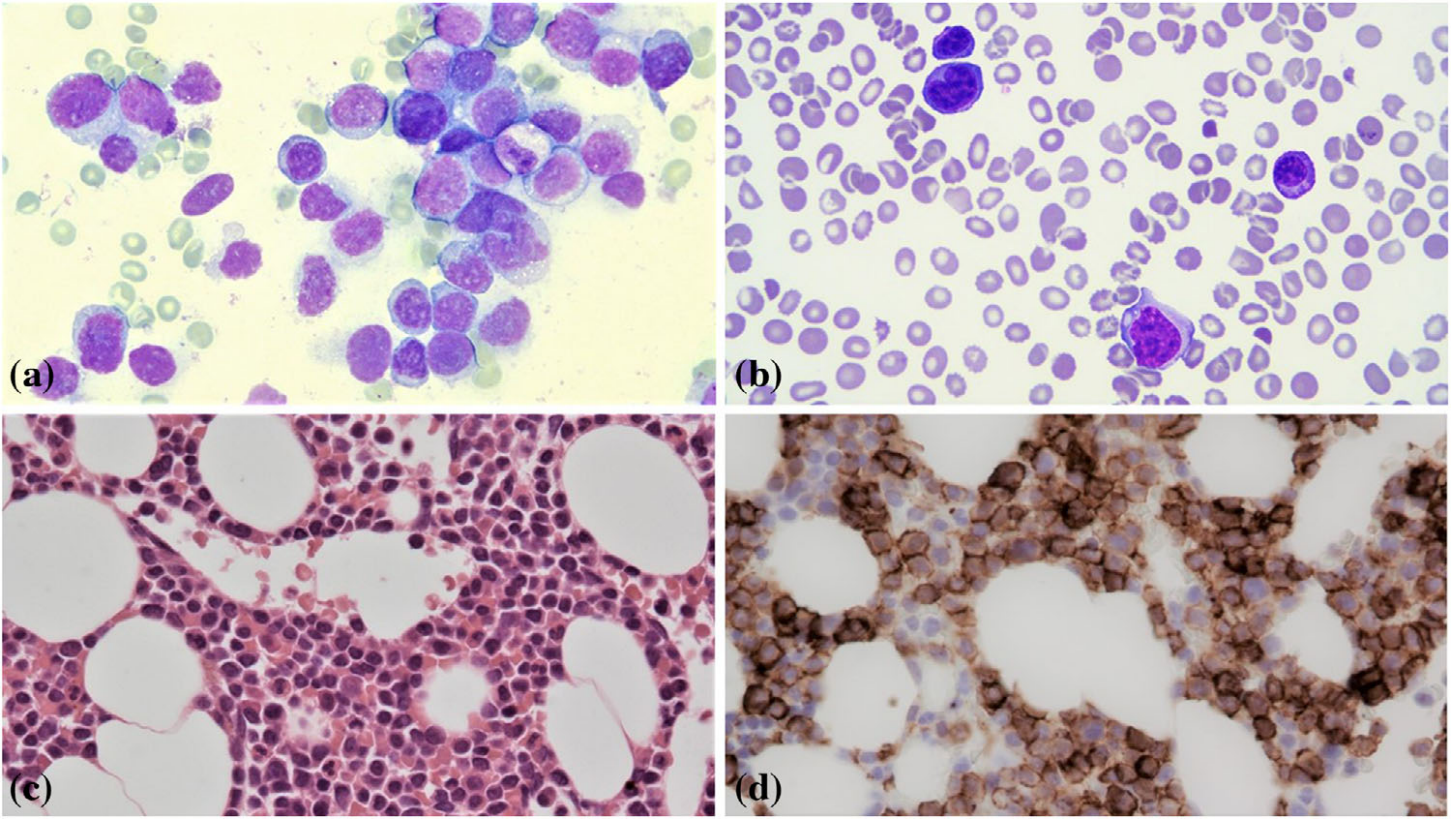
(A) Top left image, bone marrow smear (May‐Grünwald‐Giemsa stain 100x objective). The bone marrow smear was packed with medium to large‐sized blast‐like cells. (B) Top right image, blood film (May‐Grünwald‐Giemsa stain 100x objective). The blood film showed pleomorphic atypical plasma cells. (C) Bottom left image, bone marrow trephine (haematoxylin and eosin stain 40x objective). Trephine biopsy specimen was hypercellular, with an extensive diffuse interstitial infiltrate of mostly medium‐sized cells with high nuclear‐cytoplasmic ratio, coarse chromatin with sometimes small nucleoli. (D) Bottom right image (40x objective), immunohistochemistry staining of bone marrow trephine biopsy were positive for CD138

Plasmablastic myeloma is a rare and aggressive subtype with a poor prognosis. It shares nearly identical morphologic features and immunophenotypical profiles with plasmablastic lymphoma [1]. The presence of myeloma‐defining signs of hypercalcaemia, renal failure, anaemia, and bone disease in this patient, and the absence of Epstein‐Barr virus‐encoded RNA positivity support the diagnosis of plasmablastic myeloma. Knowing about this morphologic variant is also important to distinguish it from acute leukaemia. This case highlights the importance of integrated diagnosis using all diagnostic techniques.

## AUTHOR CONTRIBUTIONS

Ke Xu and Ahmed Al‐Hassani wrote the manuscript. Ke Xu, Anna Childerhouse, Ahmed Al‐Hassani and Charalampia Kyriakou critically revised the final version of the manuscript.
